# Correction: First of Its Kind Feasibility Study to Work Out the Delivery of Preventive Oral Healthcare by a Hygienist-Nurse Team During Routine Home Care Visits

**DOI:** 10.5334/ijic.10808

**Published:** 2026-03-04

**Authors:** Carrie Beltzner

**Affiliations:** 1 St. Joseph’s Health System’s Centre for Integrated Care, Canada

## Abstract

This article details a correction to: Beltzner C, 2025, First of its kind feasibility study to work out the delivery of preventive oral healthcare by a hygienist-nurse team during routine home care visits. *International Journal of Integrated Care* 25(S2):075. DOI: https://doi.org/10.5334/ijic.NACIC24075.

## Correction

The conference abstract “First of its kind feasibility study to work out the delivery of preventive oral healthcare by a hygienist-nurse team during routine home care visits” was originally published with a typo in the author’s name. While this abstract was authored by Carrie Beltzner, it was credited to Carrie Beltzer. The name has been corrected in the original article.
